# The 10th International Conference on cGMP 2022: recent trends in cGMP research and development—meeting report

**DOI:** 10.1007/s00210-023-02484-8

**Published:** 2023-04-20

**Authors:** Andreas Friebe, Jan R. Kraehling, Michael Russwurm, Peter Sandner, Achim Schmidtko

**Affiliations:** 1 Institute of Physiology, University of Würzburg, Röntgenring 9, D-97070 Würzburg, Germany; 2 Pharmaceuticals, Research and Early Development, Pharma Research Center, Bayer AG, Aprather Weg 18a, D-42096 Wuppertal, Germany; 3 Institute of Pharmacology, Ruhr-University Bochum, Universitätsstr. 150, D-44801 Bochum, Germany; 4 Institute of Pharmacology, Hannover Medical School, Carl-Neuberg-Str. 1, D-30625 Hannover, Germany; 5 Institute of Pharmacology and Clinical Pharmacy, Goethe University, Max-Von-Laue-Str. 9, D-60438 Frankfurt Am Main, Germany

**Keywords:** cGMP, Guanylyl cyclases, Phosphodiesterases, Nitric oxide, Natriuretic peptides, NO-GC, sGC stimulators, Riociguat, Praliciguat, Vericiguat, sGC activators, Mosliciguat, Runcaciguat

## Abstract

Increasing cGMP is a unique therapeutic principle, and drugs inhibiting cGMP-degrading enzymes or stimulating cGMP production are approved for the treatment of various diseases such as erectile dysfunction, coronary artery disease, pulmonary hypertension, chronic heart failure, irritable bowel syndrome, or achondroplasia. In addition, cGMP-increasing therapies are preclinically profiled or in clinical development for quite a broad set of additional indications, e.g., neurodegenerative diseases or different forms of dementias, bone formation disorders, underlining the pivotal role of cGMP signaling pathways. The fundamental understanding of the signaling mediated by nitric oxide-sensitive (soluble) guanylyl cyclase and membrane-associated receptor (particulate) guanylyl cyclase at the molecular and cellular levels, as well as in vivo, especially in disease models, is a key prerequisite to fully exploit treatment opportunities and potential risks that could be associated with an excessive increase in cGMP. Furthermore, human genetic data and the clinical effects of cGMP-increasing drugs allow back-translation into basic research to further learn about signaling and treatment opportunities. The biannual international cGMP conference, launched nearly 20 years ago, brings all these aspects together as an established and important forum for all topics from basic science to clinical research and pivotal clinical trials. This review summarizes the contributions to the “10th cGMP Conference on cGMP Generators, Effectors and Therapeutic Implications,” which was held in Augsburg in 2022 but will also provide an overview of recent key achievements and activities in the field of cGMP research.

## History of cGMP meetings with major findings presented

Originally, the 10th cGMP conference was planned to be held in June 2021. Due to the devastating pandemic, travel restrictions, and shutdowns all over the world, the conference had to be postponed for one year to June 2022. Therefore, the whole cGMP community was eagerly looking forward to personally meet and exchange again after so many months of virtual meetings. As a result, more than 100 participants joined the meeting in the old town of Augsburg in Germany.

**Fig. 1:**
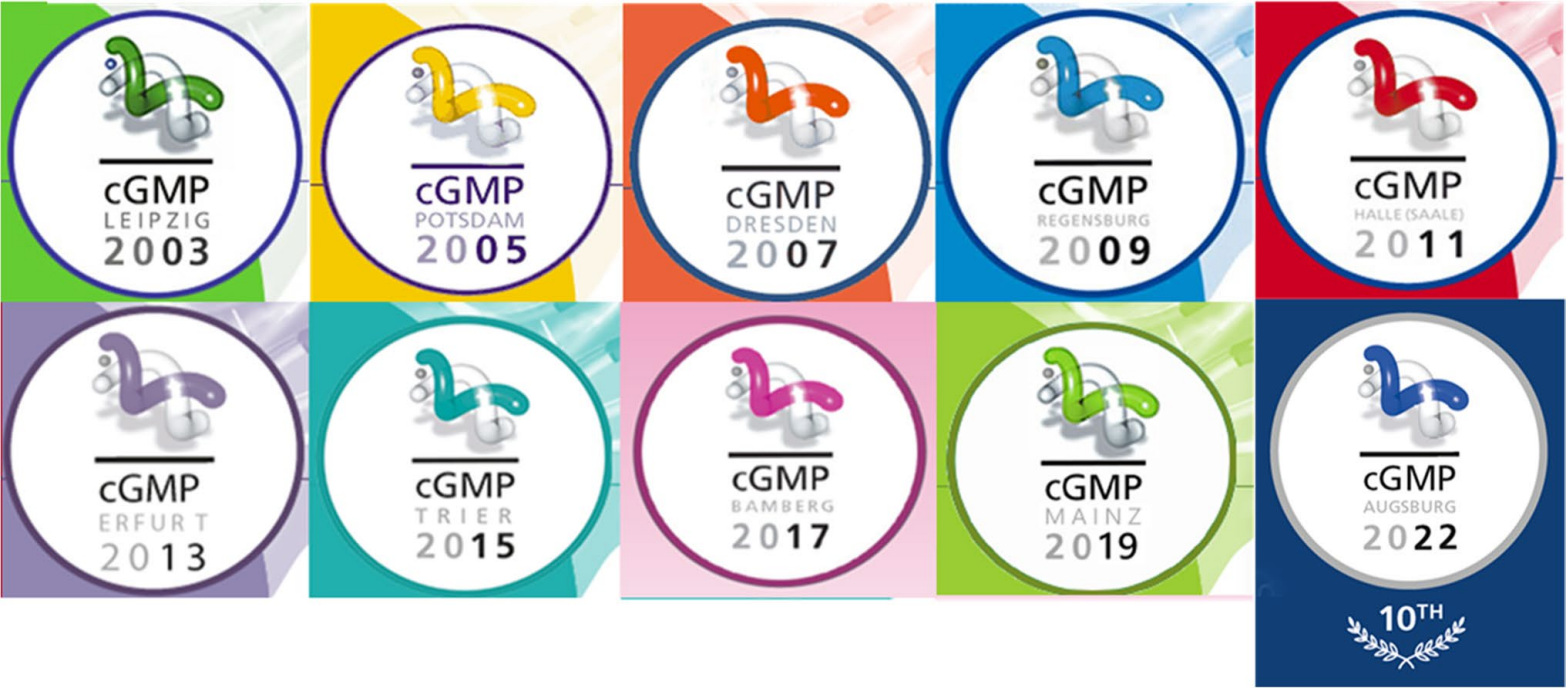
History of the International Conference on cGMP: Generators, Effectors and Therapeutic Implications. The meeting series started in Leipzig in 2003, followed by Potsdam (2005), Dresden (2007), Regensburg (2009), Halle (Saale) (2011), Erfurt (2013), Trier (2015), Bamberg (2017), and Mainz (2019). The meeting is held every second year, but the 2021 meeting had to be postponed due to the COVID19 pandemic and was now held in 2022 in Augsburg

**Fig. 2:**
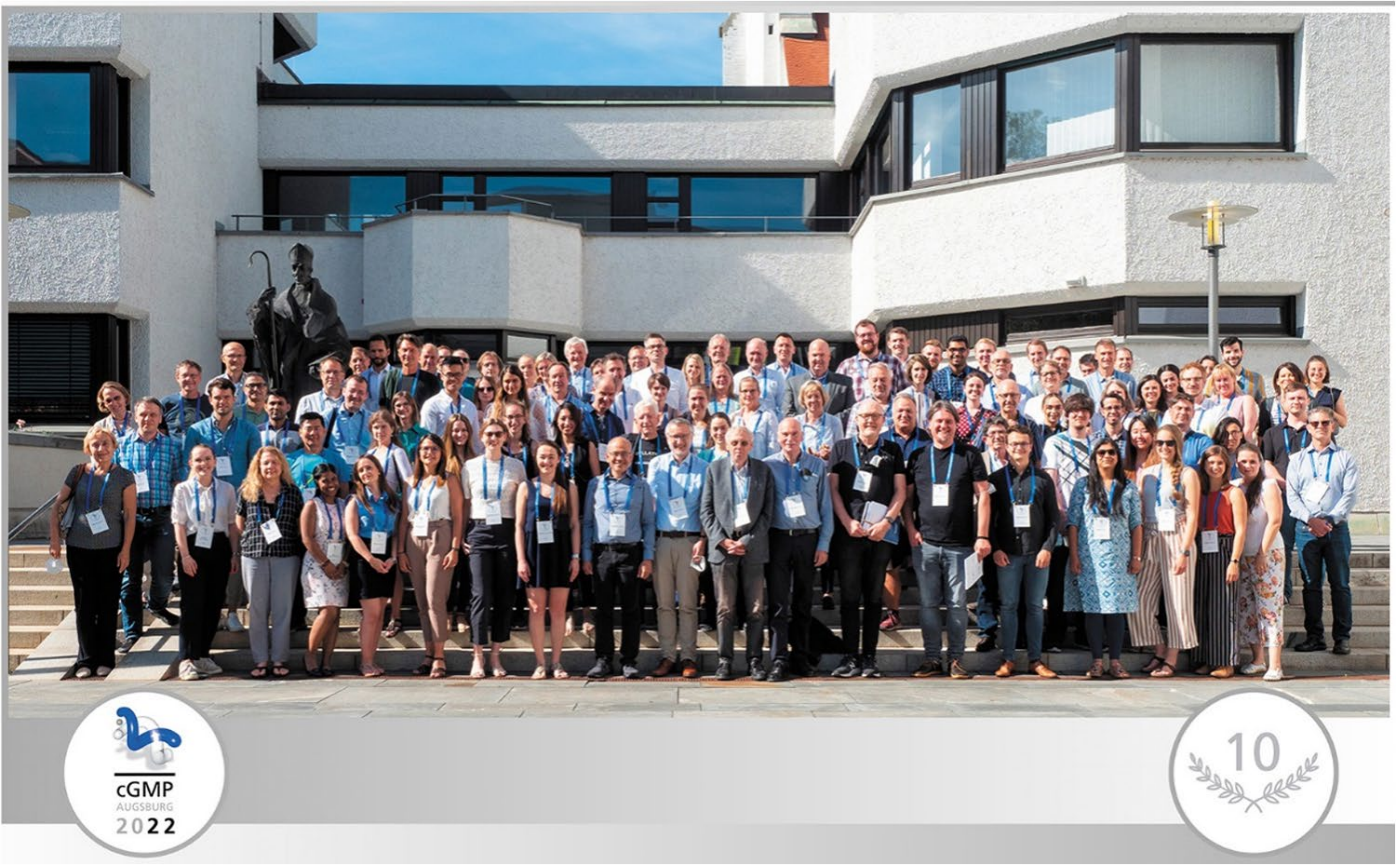
Participants of the 10th International Conference on cGMP, held in Augsburg, Germany, from June 17–19, 2022. For program and meeting information see https://www.cyclicgmp.net/index.html; For abstracts, please reach out to J Transl Med 2022 (online publication estimated for Dec 2022)

This meeting series started in 2003 with the first meeting held in Leipzig (Fig. 1). It has been held biannually and developed in this time to a major event in cGMP research since it unifies very early basic research aspects on cGMP signaling including cGMP formation and downstream targets, but also includes translational and therapeutic applications including late-stage clinical development programs. The meeting in Augsburg, being the 10th, was held in a small location at the “Haus St. Ulrich” making this meeting unique and intense (Fig. 2).

## cGMP signaling pathways

The cGMP signaling cascade is one of the key regulators for cell and tissue homeostasis. Dysregulation of the cGMP pathway leads to a plethora of diseases. cGMP can be generated by two different types of guanylyl cyclases: nitric oxide (NO)-sensitive (soluble) guanylyl cyclase (NO-GC or sGC) and transmembrane guanylyl cyclase (tmGC).

**Fig. 3:**
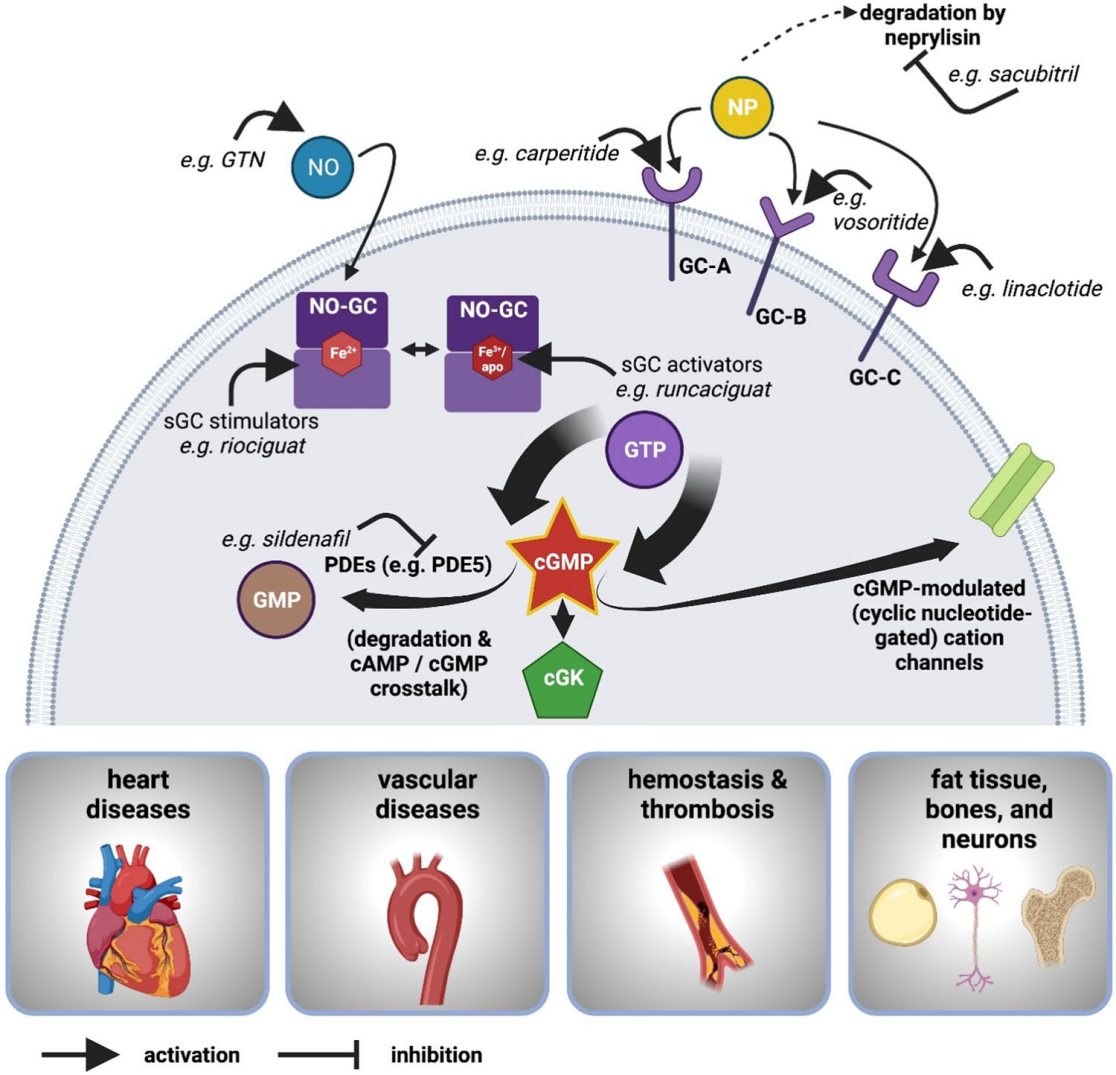
Abbreviations: cAMP, cyclic adenosine monophosphate; cGK, cGMP-dependent protein kinase; cGMP, cyclic guanosine monophosphate; GC, guanylyl cyclase; GC-A, guanylyl cyclase A; GC-B, guanylyl cyclase B; GMP, guanosine monophosphate; GTN, glyceryl trinitrate; 5′-GTP, guanosine triphosphate; NO, nitric oxide; NO-GC, NO-sensitive (soluble) guanylyl cyclase (also sGC); NP, natriuretic peptide(s); PDE, phosphodiesterase (adapted from Lukowski and Feil, BJP 2022; created with BioRender.com)

NO is produced by three isoforms of NO synthase (NOS): neuronal NOS, inducible NOS, and endothelial NOS. NO activates its main receptor, NO-GC, which generates the second messenger cGMP. Three types of cGMP-binding proteins transduce the cellular response of the cGMP pathway (Fig. 3), cGMP-modulated, cyclic nucleotide-gated (CNG) cation channels, cGMP-regulated phosphodiesterases (PDEs), and cGMP-dependent protein kinases (cGKs): (1) One of the best studied cGMP-modulated cation channels is the CNG channel involved in phototransduction in the retina. (2) Crosstalk between cGMP and cAMP signaling is achieved through cGMP- or cAMP-regulated PDEs and also occurs at the level of cAMP-dependent protein kinases, cGKs, and hyperpolarization-activated cyclic nucleotide-gated channels. (3) One of the most important actions of cGMP in the cardiovascular system is the activation of cGMP-dependent protein kinase I (cGKI), which leads to the relaxation of smooth muscle cells through various pathways. Besides the NO-driven cGMP production, seven transmembrane tmGCs (GC-A to GC-G) generate cGMP upon stimulation by natriuretic peptides and other ligands.

Therefore, cGMP generation via NO-GC and tmGC, cGMP signaling via downstream targets and cGMP degradation have been intensively studied. A variety of excellent manuscripts and review articles summarize all these aspects including a special edition of the British Journal of Pharmacology edited by R. Feil and R. Lukowski in 2021 entitled: “Recent developments in cGMP research: From mechanisms to medicines and back” (Lukowski and Feil 2022).

## Meeting highlights

### Preclinical translation and back-translation

As in previous meetings, the 2022 meeting started with a session in which recent clinical developments and applications of cGMP-increasing drugs were discussed. In addition to the benefit for patients suffering from devastating diseases, these results are also important to constantly check and refine the preclinical concepts, the assays, and in vivo models, and to adjust them. Since cGMP-increasing drugs are available for patients, back-translation from the patients to the preclinical models has become an important part in this meeting. Due to the corona pandemic, the meeting had to be postponed for 1 year; therefore, the knowledge gain was exceptional, and this topic had to be split into two sessions. In addition, an overview of the current status of clinical developments was included in Tables 1 and 2 for both, modulators of the NO-GC and tmGC pathway, respectively.

**Table 1:** Approved and currently clinically tested modulators of NO-GC (extended and modified after Petraina et al. (2022))

Compound	Indication	Clinical trial identifier	Status
sGC modulators			
sGC stimulators			
Riociguat (BAY 63–2521)	Pulmonary hypertension (PAH, CTEPH)		Approved
	Sickle cell disease	NCT02633397	Ph 2
Vericiguat (BAY 1021189/MK-1242)	Heart failure (HFrEF)		Approved
	HFrEF	NCT05093933	Ph 3
Praliciguat (IW-1973)	Type II diabetes-hypertension	NCT03091920	Ph 2
Zagociguat (CY-6463)	Alzheimer dementia	NCT04798989	Ph 2
	Mitochondrial encephalomyopathy, lactic acidosis, and stroke-like episode (MELAS)	NCT04475549	Ph 2
MK-5475	Pulmonary hypertension (PAH)	NCT04732221	Phase 2/3
	Pulmonary hypertension PH–COPD	NCT05612035	Phase 2
sGC activators			
Runcaciguat (BAY 1101042)	Chronic kidney disease (CKD)	NCT04507061	Ph 2
	Non-proliferative diabetic retinopathy	NCT04722991	Ph 2
Mosliciguat (BAY 1237592)	Pulmonary hypertension (PAH, CTEPH)	NCT03754660	Ph 1b
BI-685509	Portal vein hypertension in liver cirrhosis	NCT05161481	Ph 2
	Chronic kidney disease (CKD)	NCT04736628	Ph 2
	Systemic sclerosis (SSc)	NCT05559580	Ph 2

**Table 2:** Approved and currently clinically tested modulators of tmGCs (extended and modified after Petraina et al. (2022))

Compound	Indication	Clinical trial identifier	Status
tmGC modulators			
Neprilysin inhibitor			
LCZ696 = sacubitril + valsartan	HFrEF, HFpEF		Approved
	Resistant hypertension	NCT04637152	Ph 2
	PH-HFpEF	NCT04753112	Ph 3
	Type II diabetes	NCT03744975	Ph 2
GC-A stimulators			
Carperitide	Acute HF		Approved
Nesiritide	Acute HF		Approved
	Hypertension	NCT02608996	Ph 1/2Ph
	Diabetes	NCT03234751	1
ANX-042	Cardiorenal syndrome	NCT03019653	Ph 1
MANP	Hypertension, metabolic syndrome	NCT03781739	Ph 1
	Hypertension	NCT04542681	Ph 1
PL-3994	HFpEF	NCT04318145	Ph 2
GC-B stimulators			
Vosoritide	Achondroplasia		Approved
GC-A/GC-B stimulators			
Cenderitide	Chronic kidney disease	NCT02359227	Ph 2
	HF-renal impairment	NCT02603614	Ph 1/2
	Myocardial infarction	NCT02071602	Ph 1
GC-C stimulators			
Linaclotide	Irritable bowel syndrome		Approved

### Neurodegenerative diseases, cognitive function, and dementia

Recent excellent examples of this preclinical-to-clinical translation are neurodegenerative diseases, dementia, and other cognitive dysfunctions which have become a growing problem in a constantly aging population. In recent years, preclinical data showed that increasing cGMP could be helpful; PDE5 inhibitors have already shown preclinical efficacy but could not be successfully translated into clinics so far (Peixoto et al. 2015). However, two talks presented preclinical evidence for sGC stimulators that also translated into early clinical efficacy. A talk summarized recent preclinical studies in which the effects of sGC stimulators and sGC activators on cognitive function were investigated (Jos Prickaerts, Maastricht). Interestingly, the sGC stimulators vericiguat and BAY-747, as well as the sGC activator runcaciguat, improved learning and memory in behavioral rat models in vivo (Nelissen et al. 2021, 2022). The underlying mechanisms are not fully clear due to the observation that not only brain-penetrant compounds (such as BAY-747 and runcaciguat) but also compounds with poor brain penetration (such as riociguat) improved the behavior. Furthermore, it remains to be elucidated if sGC stimulators can be differentiated from sGC activators since they both improved learning and memory. However, there is now growing evidence that sGC stimulators and sGC activators have potential as cognitive enhancers. Furthermore, sGC stimulators have effects on microvasculature and neuroplasticity. These data are also in line with earlier studies in which the potential of sGC stimulators was demonstrated in neuroinflammation (Correia et al. 2021b) and in preclinical models of neurodegenerative diseases (Correia et al. 2021a); sGC stimulators also improve mitochondrial function and cerebral blood flow (Buys et al. 2022, conference poster presented at this meeting). Even more striking, the first clinical data from studies with the sGC stimulator zagociguat (CY-6463, formerly IW-6463) in patients with neuropsychiatric and neurodegenerative diseases showed a significant effect on cognitive performance after 2 weeks of treatment (Chris Winrow, Cambridge). Inflammatory biomarkers were improved. and positive signals in exploratory endpoints were observed. Overall, these first clinical data confirmed preclinical effects seen with sGC stimulators including zagociguat and give hope that cognitive functions can be improved, especially in patients with MELAS syndrome (myopathy, encephalopathy, lactic acidosis, stroke) and neurodegeneration. In the future, it will also be important to better understand the structural basis for optimizing blood–brain barrier permeability. This might help to generate even more efficasious treatment approaches for neurodengenerative diseases.

### cGMPopathies: disease definition based on cGMP signaling

Our current terminology of diseases which is based on the affected organs or symptoms may not allow to adequately treat patients individually or even causally since similar organ damage and symptoms may be caused by different underlying molecular pathologies. A new concept of disease definitions departing from this classical disease terminology by focusing on the underlying pathomechanisms for defining the diseases was presented (Cristian Nogales, Maastricht). He introduced “ROS-cGMPopathies” (ROCG) as common cause and could identify hypertension and heart failure patients. He also identified other ROCG in the Estonian biobank which need to be further characterized. In the future, this approach could be helpful in stratifying patients and enabling precision therapeutic interventions and might lead to a new disease understanding (Petraina et al. 2022). Ideally, it would help to identify subpopulations of patients highly responsive to drugs.

### New sGC activators on the block

The cGMP conference is an established forum to introduce new compounds which target cGMP signaling and are suitable for clinical development. At the conference, two new sGC activators were presented. First, runcaciguat (BAY 1,101,042), a novel oral sGC activator, was introduced (Peter Sandner, Wuppertal). sGC activators target the oxidized and heme-free form of NO-GC which is preferentially formed under conditions of oxidative stress. The discovery and optimization of this new compound have been described (Hahn et al. 2021) including a broad set of preclinical in vivo data suggesting that runcaciguat may be a highly effective treatment option for chronic kidney disease (CKD) regardless of the etiology (Benardeau et al. 2021; Stehle et al. 2021). Runcaciguat was dose-dependently effective in CKD models with underlying hypertensive, diabetic, and obese etiologies. Interestingly, histological staining of kidney sections revealed a higher burden of oxidative stress in renal tissues of CKD. The oral sGC activator runcaciguat has passed clinical phase 1 and is currently in a phase 2 clinical program in patients with proteinuric CKD (NCT04507061).

Although the number of sGC stimulators and activators is constantly increasing (Sandner 2018; Sandner et al. 2021), compounds that can specifically target a single organ could be more effective and additionally reduce unwanted systemic side effects. Mosliciguat (BAY 1,237,592) was introduced as a novel inhaled sGC activator, and in vitro and in vivo studies were presented (Eva-Maria Becker-Pelster, Wuppertal). Mosliciguat was shown to activate the heme-free NO-GC and to improve cardiopulmonary circulation in minipigs and rats. Inhalation resulted in selective lung effects by reducing pulmonary artery pressure without affecting systemic blood pressure. The effects of mosliciguat showed a long duration and were enhanced under conditions of oxidative stress. Based on this, mosliciguat is tentatively aimed at group 3 patients with pulmonary hypertension in whom the ventilation/perfusion mismatch is high and other PAH drugs have failed so far. In fact, mosliciguat selectively vasodilated ventilated parts of the lung, leading to a reduction in desaturation (Becker-Pelster et al. 2022).Therefore, given its mode of action as an sGC activator and its selective application route, mosliciguat could advance NO-GC-based pharmacotherapy; phase 1b studies for pulmonary hypertension, which are currently underway, will provide more information on the future of this compound in pulmonary hypertension (NCT03754660).

**Fig. 4:**
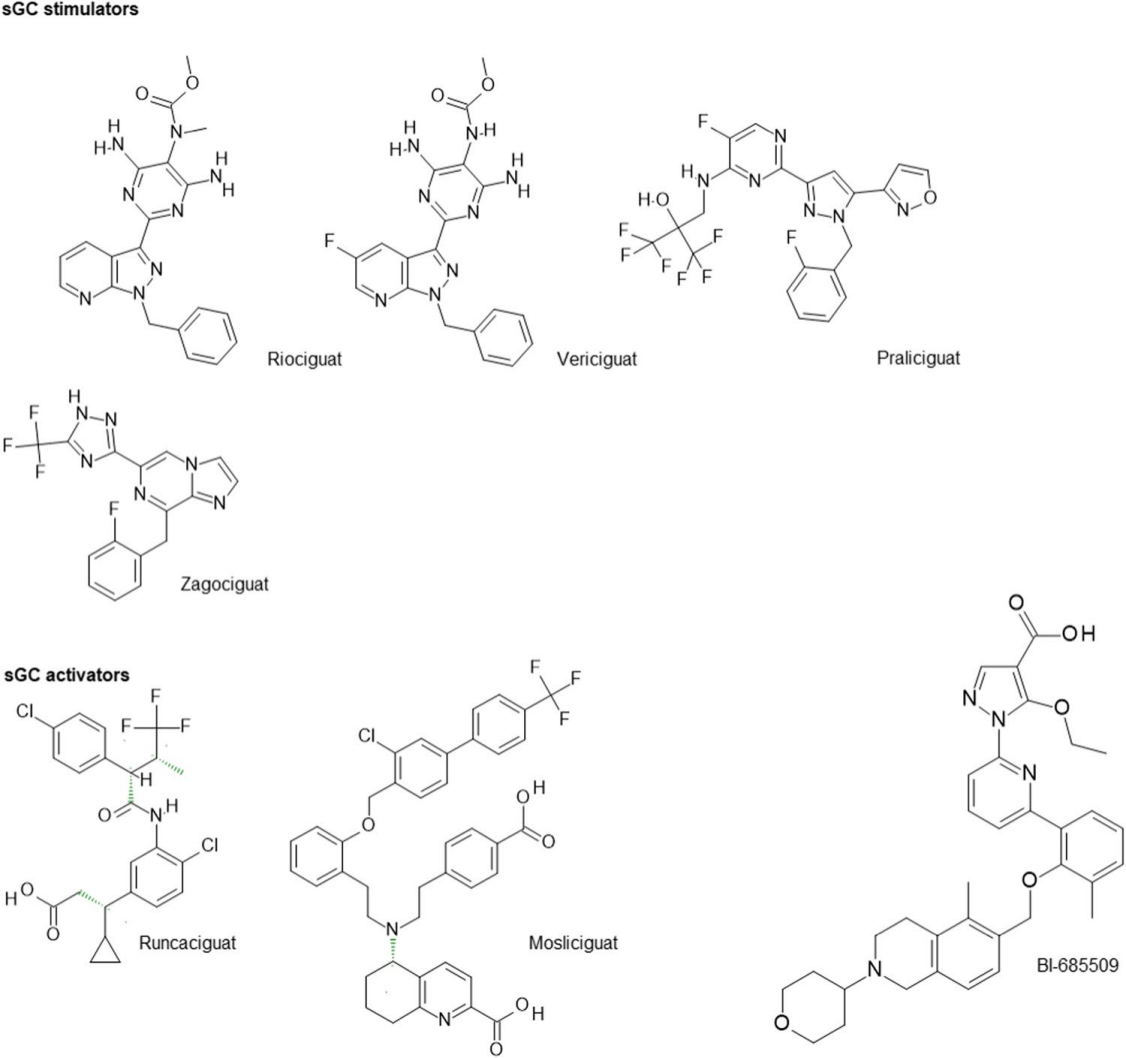
Structures of sGC-agonist compounds which are currently marketed (Riociguat and Vericiguat) or in clinical development (created with BIOVIA Draw)

Compared to sGC stimulators of which two compounds have already reached the market, research and clinical development of sGC activators lag far behind. This might be related to the limitations of previous compounds. However, with the second-generation sGC activators runcaciguat, mosliciguat, or BI-685509 which is also in clinical development (Table 1), novel opportunities are given to improve our understanding of the treatment potential of enhancing cGMP signaling under persisting oxidative stress by activating apo-NO-GC. The chemical structures of second generation sGC activators are summarized in Fig. 4 which also includes sGC stimulators presented on the meeting.

- Brain and neurosensory system

Numerous studies in the last three decades have demonstrated that NO signaling essentially contributes to fine-tuning of synaptic transmission in the central nervous system (CNS) (Garthwaite 2019; Lundberg and Weitzberg 2022). Most of the effects of NO are mediated by NO-GC, of which two isoforms (NO-GC1 and NO-GC2) exist that exhibit comparable regulatory and enzymatic properties. Accumulating evidence indicates that the two isoforms show different distribution patterns in the CNS and exert distinct functional roles (Peters et al. 2018; Petersen et al. 2019). Recent live cell fluorescence imaging of primary hippocampal neurons isolated from mice lacking NO-GC1 or NO-GC2, which were crossed with cGi-500-expressing cGMP indicator mice, revealed that NO-GC2 plays the predominant role in the generation of cGMP signals induced by *N*-methyl-d-aspartate (NMDA) and AMPA (α-amino-3-hydroxy-5-methyl-4-isoxazole propionic acid) glutamate receptors (Doris Koesling, Bochum). Furthermore, the observation that NMDA- and AMPA-induced cGMP signals are modulated by PDE inhibitors in a specific manner points to different pools of cGMP in the hippocampus, most likely in specific compartments equipped with distinct sets of PDEs (Giesen et al. 2022).cGMP also has essential functions in mammalian embryonic development (Schmidt et al. 2022). One important cGMP-dependent process is neuronal polarization, in which neurons form the distinct compartments of the dendrite and the axon. Recent data demonstrated that apical dendrite development is directed by spatially organized extrinsic cues and mediated by a cGMP synthesizing complex composed of NO-GC that associates with the scaffolding protein scribble and the semaphorin3A co-receptor plexinA3 (Maya Shelly, Stony Brook). The associations within the complex are not only necessary for semaphorin3A-mediated cGMP increases in dendrites and CA1 apical dendrite development, but also for bipolar polarity establishment in CA1 pyramidal progenitors. Together, these findings demonstrate that apical dendrite development is promoted by the extrinsic Sema3A/PlexinA3 cue and mediated by a localized cGMP-synthesizing complex (Szczurkowska et al. 2022).

Two new studies shed light on the allosteric gating of cyclic nucleotide-gated (CNG) channels, which convert light- and odor-induced changes in intracellular cyclic nucleotides into electrical signals and are essential for vision and smell (Michalakis et al. 2018; Barret et al. 2022). Various mutations in CNG channels cause retinitis pigmentosa and achromatopsia, but the underlying pathogenic mechanisms are poorly understood. Cryo-electron microscopy structures of human and nematode CNG channels have been recently reported (Jian Yang, New York). These studies revealed that mutated human CNGA3_R410W/CNGB3 and nematode TAX4_R421W channels are spontaneously active without cGMP and induce cell death, pointing to cone degeneration triggered by spontaneous CNG channel activity as a possible cause of achromatopsia (Zheng et al. 2022a, b).

Recent work uncovered an unexpected function of cGMP in cochlear synaptopathy, i.e., dysfunction or loss of afferent auditory fibers that can progress with aging or following non-traumatic loud sound (Morgan Hess, Tübingen). A study in mice revealed that central neural responses to age-related cochlear synaptopathy may differ depending on individual stress responses. Acute high stress induction can reduce increased cochlear and central auditory responses, and stress-induced decrease in central compensation can be restored by elevating cGMP levels with a PDE9 inhibitor. These data suggest that the successful adjustment to cochlear synaptopathy is a cGMP- and glucocorticoid-dependent process (Savitska et al. 2022).

(b) Clinical translation/cardiovascular

Starting with the early descriptions of the use of nitrates in patients with angina pectoris in the nineteenth century and the discovery of NO in the cardiovascular system, which was awarded the Nobel prize in 1998, no other area of disease is so closely linked to cGMP (Monica et al. 2016). In fact, at least four drug approvals further strengthened this link: (1) the PDE5 inhibitors for erectile dysfunction and pulmonary arterial hypertension (PAH); (2) the first sGC stimulator in class, riociguat, in pulmonary arterial hypertension; (3) more recently the neprylisin inhibitor sacubitril combined with valsartan as well as (4) the sGC stimulator vericiguat in chronic heart failure (HF). However, there are still various questions in regard to mode of action, therapeutic potential in cardiovascular diseases beyond PAH and HF, but also differentiation of tmGC- and NO-GC-related pathways and their impact on cardiovascular health and disease is still heavily investigated (Emdin et al. 2020).

### “Lumpers and splitters” in heart failure treatment

At the beginning of the session, by talking about “lumpers and splitters,” an excellent overview of the legacy and future of the treatment of heart failure was presented (Thomas Lüscher, London). It was illustrated how in the beginning and still today, the definition of large patient populations (“lumpers”) helped to advance treatment approaches. More recently and based on a steadily advancing understanding of the genetic background, especially also for cardiomyopathies, the time for “splitting” might allow for more personalized treatment approaches and a massively increased treatment effect in these specific subgroups.

### Clinical efficacy of vericiguat in chronic heart failure with HFrEF

In recent years, a new sGC stimulator, vericiguat (BAY 1,021,189), was discovered and intensively profiled in preclinical models for cardiovascular disease and heart failure (Follmann et al. 2017). This compound was then clinically developed in phase 2 studies in patients with chronic heart failure (Pieske et al. 2014) in both HFrEF [SOCRATES reduced trial; (Gheorghiade et al. 2015)] and HFpEF [SOCRATES preserved trial (Pieske et al. 2017)]. Based on these results, vericiguat was studied in a pivotal phase 3 study, the VICTORIA study in HFrEF (Armstrong et al. 2018). At the conference, the results of the VICTORIA study conducted on 5050 patients with heart failure with reduced ejection fraction (HFrEF) who had a recent worsening HF event were presented (Paul Armstrong, Edmonton). Vericiguat demonstrated a clinically relevant and statistically significant reduction in the primary composite endpoint of cardiovascular death and hospitalization for heart failure. Based on these results, in 2021, vericiguat was approved for the treatment of patients with HFrEF (Markham and Duggan 2021) and is therefore the second sGC stimulator after riociguat available for patients.

### Clinical efficacy of praliciguat in female heart failure patients with HFpEF

Interestingly, both Bayer and Cyclerion conducted studies with the sGC stimulators vericiguat and praliciguat in chronic heart failure patients with preserved ejection fraction (HFpEF): the Vitality HFpEF study with vericiguat (Butler et al. 2019) and the Capacity HFpEF study with praliciguat (Udelson et al. 2020). In both studies, neutral and no significant beneficial effects of the drugs were observed (Armstrong et al. 2020, Udelson et al. 2020). However, a post hoc analysis of the capacity HFpEF trial with praliciguat showed effectiveness in female patients. In the subgroup of postmenopausal women, praliciguat treatment led to a clinically significant improvement in peak oxygen consumption (VO_2_) compared to placebo and increased ventilatory efficiency, peak workload, and distance in the 6-min walk test. Although these were post hoc analyses, elderly, postmenopausal women with HFpEF may benefit from NO-GC stimulation and prospective clinical trials in this population are warranted (Andreas Busch, Cambridge). Interestingly, these data are in line with clinical studies with ARNI treatment (Sacubitril-Valsartan combination) as seen in the Paragon Trial with Entresto® (McMurray et al. 2020). In summary, these clinical data suggest that especially women with HFpEF are profiting from cGMP increasing drugs of both sGC stimulators and stimulation of NP pathways. Currently, the understanding of this difference is limited and speculative. However, the comorbidities in conjunction with estradiol decline in the postmenopausal age might impair cGMP signaling to a higher extent in female patients (Sabbatini and Kararigas 2020). Future research is necessary to better understand these sex-specific treatment effects of cGMP-enhancing drugs in HFpEF also to better tailor cGMP-enhancing therapies for the female HFpEF patient population.

### Arrhythmias and natriuretic peptides

Arrhythmias and atrial fibrillation (AF) are still fundamental medical problems and difficult to treat. Various treatment approaches have failed, and nonclinical in vivo models often miss translatability to patients. Although it is known that the gene *NPPA*, encoding for atrial natriuretic peptide (ANP), is linked to familial AF, the underlying cellular mechanisms by which mutations in this gene cause AF remain unclear. Recently, it was shown that the NPPA-S64R mutation reduced the functional activity of the electron transport chain and the overall cellular oxidative capacity in human iPSC-derived atrial cardiomyocytes. The NPPA gain-of-function mutation also enhanced the expression and function of a cardiac potassium channel and shortened the atrial action potential duration (Ly et al. 2022). These data offer the opportunity for personalized therapies targeting cardiomyocyte metabolism or ANP signaling in patients carrying the NPPA mutation (Dawood Darbar, Chicago).

### Genetic variants of cGMP signaling and cardiovascular diseases

Genetic sequencing and genome-wide association studies (GWAS) analysis have made tremendous progress in recent years. Genetic variants in more than 200 genes have been shown to be associated with coronary atherosclerosis and myocardial infarction. Importantly, several variants are located in genes that are involved in cGMP signaling (Leineweber et al. 2017; Wobst et al. 2018). Recently, genetic downregulation of NO-cGMP signaling was found to increase the risk for atherosclerosis and myocardial infarction. Genetic analyses and in vitro and in vivo experimentation were used to investigate the functional role of genetic variants in genes that are involved in cGMP signaling in atherosclerosis. Rare coding variants in genes encoding NO-GC and PDE5 were associated with coronary artery disease and atherosclerosis. These findings suggest a functional role of genetic variations in NO-GC and PDE5 in platelets and vascular smooth muscle cells which are associated with coronary artery disease and myocardial infarction. Since these genes are crucially involved in cGMP signaling, these findings highlight the importance of this pathway in atherosclerosis and the harmful consequences could potentially be prevented by cGMP increasing pharmacological mechanisms (Torsten Kessler, Munich).

A fine-tuned interplay between metabolism, oxidative stress, inflammation, and cardiovascular diseases including heart failure exists. In a plenary lecture, an excellent insight into the metabolic and redox dysregulation in heart failure was provided (Christoph Maack, Würzburg). Although these mechanisms are only understood in part, they might offer future heart failure therapies with enhanced efficacy.

(iii) Cardiovascular diseases and cGMP

It is still a matter of debate how cGMP on a cellular and molecular level improves heart function and is beneficial in cardiovascular diseases. Therefore, intense research efforts are ongoing to better understand the cellular targets as well as the downstream regulation and compartmentalization. One talk focused on the redox regulation of cGMP-dependent protein kinase I (cGKI), which has been reported to result in disulfide bonds between cysteines 42 of the dimerizing subunits and between cysteines 117 and 195 (near/in the cGMP binding sites) within the subunits after treatment with experimental nitroxyl donors [Angeli’s salt, 1-nitrosocyclohexyl acetate (NCA)] and nitroxyl donors developed as drugs for the treatment of acute decompensated heart failure [CXL-1020 and cimlanod (CXL-1427)]. To analyze the impact of the intra(subunit) disulfide bond in physiological and pathophysiological conditions, a knock in mouse line with a Cys195 to Ser mutation was generated incapable of forming the intra-disulfide bond 117–195. These mice showed a gastrointestinal phenotype with enlarged caecum and increased intestinal transit time, reminiscent of knockout mice of NO-GC or cGKI. However, blood pressure assessed by telemetric recordings was unchanged. Furthermore, in (untreated) KI and wild-type (WT) animals, no blood pressure effect of CXL-1020 at a dose of 9 mg/day was observed. On the contrary, micromolar concentrations of CXL-1020 induced transient relaxation of isolated mesenteric arteries in both genotypes; inhibition of the relaxation by the NO-GC inhibitor ODQ indicated activation of NO-GC rather than direct targeting cGKI as underlying mechanism. Interestingly, angiotensin II treatment increased blood pressure in WT and KI in a similar way; however, CXL-1020 induced a reduction in blood pressure by 10 mmHg only in WT (Kamynina et al. 2022). Thus, intra-disulfide formation within cGKI subunits is capable of differentially reducing blood pressure in hypertensive but not healthy animals (Friederike Cuello, Hamburg).

Mixed lineage kinase 3 (MLK3) has recently been shown to interact with cGKI as its substrate and to mediate the therapeutic effects of cGMP elevation on left ventricular pressure overload (transverse aortic constriction (TAC)) apparently via the nuclear factor transcription factor of activated T cells (NFAT). Global MLK3 knockout mice displayed elevated blood pressure; since acute hypotensive effects of PDE5 inhibition or NO-GC stimulation were preserved, MLK3 does not mediate cGMP effects on blood pressure (Calamaras et al. 2021). MLK3 harbors a CRIB domain (Cdc42/Rac interactive binding) that mediates the activation of MLK3 by Cdc42 (Robert Blanton, Boston). A mutant mouse line with point mutations in CRIB that disrupts Cdc42 activation of MLK3 displayed increased blood pressure, left and right ventricular hypertrophy, and reduced expression of MLK3 in the left ventricle; in moderate TAC, only mutant mice showed reduction in left ventricular function. Thus, activation of MLK3 via the CRIB domain is required for its function. To specifically analyze the function of MLK3 in cardiac myocytes independently from vascular effects, a cardiac myocyte-specific MLK3 knockout was created. As expected, blood pressure was unaffected, but a small reduction of left ventricular function was apparent; furthermore, female mice displayed an increased end-diastolic diameter (Liu et al. 2022).

Heart failure patients with preserved ejection fraction (HFpEF) often display marked obesity, and sarcomere function in obese patients with HFpEF is substantially depressed compared to non-obese patients with HFpEF (Aslam et al. 2021). Thus, pharmacological treatments for obesity are desirable. cGMP has been implicated in regulating energy expenditure, but most cGMP-elevating drugs simultaneously decrease blood pressure. Consequently, a recent study focused on PDE9 as inhibition of this cGMP-specific PDE has been implicated to affect energy homeostasis but not to reduce blood pressure. In a mouse model of diet-induced obesity with and without mild transverse aortic constriction (TAC), inhibition of PDE9 decreased body weight and reduced total fat mass, blood glucose, triglycerides, and cholesterol, as well as liver mass (Mishra et al. 2021). Interestingly, these effects occurred without changing food intake and activity but by increasing O_2_ consumption and CO_2_ production. Accordingly, flag-tagged PDE9 was found in cardiac mitochondrial fractions, and GFP-labelled PDE9 was found to localize to cardiac mitochondria as assessed by immunogold where it increased basal and maximal O_2_ consumption in a cGMP kinase-dependent manner. Apparently, PPARɑ mediates the effect of PDE9 inhibition because PPARɑ inhibitors did not affect body weight and fat mass per se but abolished the effect of PDE9 inhibition on these parameters. In adipocytes, PDE9 inhibition augmented ANP-induced lipolysis which was mediated by PPARɑ. Interestingly, the metabolic effects of PDE9 inhibition are restricted to males and ovariectomized females; non-ovariectomized females were not affected because estrogen receptor activation impeded PPARα DNA binding and downstream regulation of genes involved in fatty acid metabolism. In summary, these data suggest expanding PDE9 inhibitor treatment to men and postmenopausal women with obesity (David A. Kass, Baltimore).

A previous study demonstrated the requirement of NO-GC in cardiac myocytes for the reduction in infarct size induced by post-ischemic application of PDE5 inhibitors or sGC activators (Frankenreiter et al. 2018). However, whether ischemia or reoxygenation affect the cGMP cascade remained unknown. This question was investigated in a recent study in which isolated adult murine cardiac myocytes subjected to hypoxia displayed reduced cGMP FRET responses to CNP + IBMX, consistent with the assumption of increased basal cGMP; furthermore, hypoxia increased cGMP (determined by radioimmunoassay) (Bork et al. 2021). Mechanistically, this is explained by reduced levels of PDE3 in cardiomyocytes as assessed in Western blots and corroborated by abolished effects of PDE3 inhibition seen in cGMP FRET measurements. As the response to hypoxia was not different between WT controls and cardiomyocyte-specific NO-GC knockout, the increased basal cGMP in cardiomyocytes is unlikely to be formed by cardiomyocyte NO-GC (Viacheslav O. Nikolaev, Hamburg).

Another talk at the cGMP conference reported on the effects of CNP on cardiac function. In general, the effects of CNP on cardiomyocyte function are different from those elicited by ANP, although both peptides increase cardiomyocyte cGMP. By activating GC-B, CNP elicits lusitropic and negative inotropic responses via phospholamban and troponin I phosphorylation. Furthermore, CNP reduces myocyte stiffness via titin phosphorylation (Manfra et al. 2022). A HFpEF mouse model was generated by feeding a high-fat diet in combination with NO synthase inhibition (L-NAME). In these HFpEF mice, CNP reduced cardiomyocyte cross-sectional area and end-diastolic and end-systolic pressures (Dulasi Arunthavarajah, Oslo).

(iv) Other indications and non-canonical signaling

Given the broad expression of NO-GC and tmGC in various tissues, it is obvious that cGMP signaling exerts significant functions beyond the cardiovascular, cardiopulmonary, and cardiorenal tissues. Furthermore, the in vivo relevance of non-canonical cNMPs like cCMP and cUMP in cells and tissues is becoming better understood.

### cGMP and adipose tissue

Various studies have revealed that cGMP is important for the regulation of adaptive thermogenesis (Reverte-Salisa et al. 2019; Zhang et al. 2021), and accumulating data indicate that PDE9 contributes to cGMP hydrolysis in adipocytes. Previous work demonstrated that PDE9 expression in brown adipocytes is decreased after cold exposure or treatment with β-adrenergic receptor agonists. A recent study found that obesity induced by a high-fat diet was reduced in PDE9 knockout mice (Sheila Collins, Nashville). Specifically, the loss of PDE9 increased the respiratory capacity of brown adipose tissue, modestly increased global energy expenditure, and resulted in an associated decrease in weight gain. Hence, PDE9 is a regulator of energy metabolism, and its inhibition may be a new strategy for treatment of obesity (Ceddia et al. 2021).

### cGMP and retinopathies

Phototransduction in the retina relies on cGMP signaling, and some of the inherited retinopathies, which may lead to severe visual impairment or blindness, are caused by defects in genes encoding cGMP effectors (Tolone et al. 2019). Recent progress has been made in the treatment of retinopathies caused by mutations in cyclic nucleotide-gated channel subunits or PDE6 isoforms (Stylianos Michalakis, München). Among the most promising options are gene-specific approaches using adeno-associated virus-based vectors to express a healthy version of the disease-causing gene in affected cells of a patient (Pavlou et al. 2021). A recombinant adeno-associated virus (rAAV) vector allowing rod-specific expression of human CNGB1 (rAAV5.hCNGB1) demonstrated efficacy in CNGB1 knockout mice (Wagner et al. 2021b). Moreover, gene therapy of achromatopsia caused by mutations in the CNGA3 gene has already reached the clinical phase, and a trial with subretinal AAV8.CNGA3 gene therapy in nine patients with CNGA3-linked achromatopsia demonstrated a good safety profile over 3 years (Reichel et al. 2022). Several clinical trials with vector-mediated gene therapy of inherited retinopathies are in progress (Michalakis et al. 2022).

### cGMP and bone growth

In the bone, NO/cGMP/cGK signaling positively regulates osteoblast proliferation, differentiation, and survival, and cGMP-elevating agents have bone-anabolic effects in mice (Kim et al. 2021). In a recent study, osteoblast-specific cGK2 knockout mice have been generated (Renate Pilz, San Diego). This mouse line exhibited an unexpected sexual dimorphism: Male mice had fewer osteoblasts, reduced bone formation rates, and lower bone volumes, whereas no bone abnormalities were observed in female mice. Moreover, expression of genes involved in osteoblast differentiation and Wnt/β-catenin signaling was lower in primary osteoblasts and bones of male but not female knockouts, suggesting that cGK2 drives bone acquisition via the Wnt/β-catenin pathway in male mice (Kalyanaraman et al. 2022).

### cGMP and Marfan syndrome

Marfan syndrome (MFS) is an autosomal dominant connective tissue disorder. Data from a recent publication indicate that NO-cGMP signaling is involved in the pathology of MFS (de la Fuente-Alonso et al. 2021). Patients with MFS show vasculopathy with problems in aortic remodeling, including aneurysms, stenosis, and atherosclerosis. In fact, 1 in every 3000–5000 MFS patients is affected by aortic aneurysm and dissection (Pearson et al. 2008). In patients with MFS, supraphysiological levels of NO occur that may induce dilation of the aorta in the thoracic aortic aneurysm and may ultimately lead to aortic dissection. In fact, plasma cGMP levels in these patients are increased, as is the nitrated plasma index. Elevated NO was also shown in an MFS mouse model (expressing a mutated form of fibrillin-1, a component of extracellular microfibrils that provides a scaffold for the formation of elastic fibers) leading to high levels of eNOS (Oller et al. 2017) which resulted in increased levels of nitrated proteins in plasma and aortic tissue. Consequently, activated NO-GC/cGK signaling increased plasma levels of cGMP and aortic pVASP-S239 staining. In these mice, NO donors were shown to induce aortopathy, which was reverted by inhibition of NO-GC or cGK. From these data, it is obvious that cGMP signaling is a potential target for the therapy of MFS disease (Juan M. Redondo, Madrid).

### cCMP and cUMP in bacteria

Besides cAMP and cGMP, the cyclic pyrimidines 3′,5′-cyclic cytidine monophosphate (cCMP) and 3′,5′-cyclic uridine monophosphate (cUMP) which are formed by specific pyrimidine nucleotide cyclases have been detected in multiple organisms and cell types (Seifert and Schirmer 2022). However, in contrast to cGMP and cAMP, the biological role of cyclic pyrimidines has remained poorly understood. One talk reported that cCMP and cUMP act as immunity signaling compounds in bacteria (Benjamin Morehouse, Boston). cCMP and cUMP are synthesized by bacterial pyrimidine cyclase enzymes in response to phage infection and activate immune effectors that execute an antiviral response. These defense systems encoding pyrimidine cyclases, referred to as Pycsar (pyrimidine cyclase system for antiphage resistance), are widespread in prokaryotes (Tal et al. 2021). It remains to be determined whether cCMP and cUMP mediate antiviral defense in other organisms or play roles outside of immunity.

### cGMP in Dictyostelium

Another talk addressed the function of cGMP in cell movement and chemotaxis in Dictyostelium, a eukaryotic unicellular organism that moves like neutrophils with pseudopodia (Peter van Haastert, Groningen). Research over the past decades uncovered that two G protein-regulated guanylyl cyclases, a large cGMP target protein, and a cGMP-stimulated PDE form a protein complex in Dictyostelium. cGMP produced in the protein complex mediates the formation of branched F-actin in the front of the cell, and it promotes myosin filament formation and pseudopod inhibition in the rear of the cell, resulting in cell movement and chemotaxis (van Haastert et al. 2021).

(e) NO-GC structure, modulators, and function

Despite the resolution of the full-length NO-GC, which was presented on the last cGMP conference in 2019 (Horst et al. 2019; Kang et al. 2019), several questions on cGMP formation and NO-GC function are still remaining, including heme insertion or modulator binding and activity (Liu et al. 2021). Several recent reviews nicely introduce to NO-GC regarding signaling, pharmacological modulation, and the resulting effects (Hofmann 2020; Wittenborn and Marletta 2021; Sumi and Ghosh 2022; Wu et al. 2022).

At the 6th cGMP conference 2013, the mechanism of heme insertion into NO-GC by the chaperone hsp90 was first reported (Ghosh et al. 2014). Subsequent studies on heme insertion in NO-GC were presented at the 2022 meeting (Dennis J. Stuehr, Cleveland): In the current model, a major portion of NO-GC is thought to exist in the heme-free form. Under these conditions, the β1 subunit of NO-GC is complexed with chaperone hsp90 which prevents heterodimerization with the α1 subunit. The delivery of heme to the β1 subunit is dependent on GAPDH; after heme insertion, hsp90 falls off and allows β1 to interact with α1, thus forming an active and NO-sensitive heterodimer. In fact, low levels of NO enhance heme insertion, whereas high levels of NO inhibit this process (Dai et al. 2022). The latter assumption is thought to explain why more heme-free NO-GC is found in severe asthmatics. According to the model, heme-free heterodimer does not exist, a statement that was highly discussed and appears implausible based on a study with a heme-free knock-in mutant (Thoonen et al. 2015).

The role of NO-GC2, i.e., the heterodimer made up of the α2 and β1 subunits, is still enigmatic. NO-GC2 does not differ from NO-GC1 (which is expressed much more frequently in most tissues) in terms of its biochemical properties [NO activation or enzyme activity (Russwurm et al. 1998)]. NO-GC2 is believed to be localized in the cytosol and associated with plasma membranes through a specific C-terminal amino acid sequence (Russwurm et al. 2001). The isoform is expressed in the nervous system to a similar degree as NO-GC1, but is also found in the lung and, to a lesser degree, in other organs (Mergia et al. 2003).

The potential role of the α2 subunit as a candidate cancer gene was already presented and debated in the literature in 2006 (Sjoblom et al. 2006; Rubin and Green 2007). At this meeting, overexpressed NO-GC2 but not NO-GC1 was shown to be located at cell–cell contacts in HEK293 cells which implies a role in regulating cell adhesion and cell–cell contacts (Soenke Behrends, Braunschweig). A mutated form of NO-GC2 (carrying a mutation in the α2 subunit originally identified in a patient with lung adenocarcinoma) was overexpressed in HEK293 cells and found not to bind to cell–cell contacts (Hochheiser et al. 2016). Loss of cell–cell contacts could interfere with NO-GC2’s signaling properties, which could affect cancerogenesis. Whether the mutation in NO-GC2 is coincidental or in fact a driver mutation might be shown in the future.

The concept of NO binding to the heme of NO-GC is multifaceted in its exact mechanism, but the interaction and consecutive conformational changes are accepted as mechanisms of activation. Much less is known about the additional effects of NO via non-heme interaction. Nitrosation of NO-GC has been shown to occur (Sayed et al. 2007) in response to oxidative stress at up to 10 sites, and their solvent exposure indicates possible protein–protein interactions (Beuve et al. 2016). In fact, nitrosation is thought to result in unresponsiveness of the enzyme to NO. New data (Cui et al. 2022) presented in Augsburg (Annie Beuve, Newark) indicate that under oxidizing conditions, NO-GCα1 itself mediates transnitrosation, i.e., the specific transfer of its nitrosothiols to more than 200 proteins in cardiac and smooth muscle cells. Thioredoxin1 was identified as one of the transnitrosation targets of NO-GC. A specific effect was shown on the small GTPase RhoA, which was inhibited after nitrosation. RhoA nitrosation required both NO-GCα1 as a nitrosothiol donor and thioredoxin1 as a nitrosothiol relay. The proof of NO-GC-thioredoxin-RhoA signaling was shown in oxidized cells that were depleted for NO-GCα1: In these cells, RhoA activity was increased and SNO-RhoA levels decreased. Thus, a non-canonical signaling pathway independent of cGMP involving thioredoxin1-mediated transnitrosation to regulate RhoA activity is conceivable. Further work needs to be done to show the physiological relevance of this putative signaling cascade.

(f) Emerging topics

cGMP and cAMP have been shown to induce “browning” of adipose tissue and to enhance lipolysis, thermogenesis, and energy expenditure. In a talk on the cGMP conference, results of a recent study with FRET-based analysis of cGMP generation and degradation in brown adipocytes (BA) derived from mice expressing the biosensor cGi-500 were reported (Daniel Rowland, Bonn). In predifferentiated cells, CNP-activated GC-B was the prevalent cGMP source and CNP-induced cGMP was degraded by PDE1 and PDE3 and not by PDE9. The lower, but detectable NO-induced cGMP was mainly hydrolyzed by PDE3 and PDE9, findings that suggest locally distinct cGMP pools. In mature BA, increased NO-induced cGMP signals became detectable and now PDE2 and PDE3 were identified as the responsible PDEs. With the help of a Gq-coupled DREADD receptor (designer receptor exclusively activated by designer drugs) causing a to increase intracellular Ca^2+^, PDE1 was activated reduction of the CNP-induced cGMP signal, thereby demonstrating a crosstalk between Ca^2+^ and cGMP formed by GC-B. Thus, PDE1 provides a link for modulatory effects of Ca^2+^ on BA differentiation.

Uroguanylin, the activator of GC-C, has been implicated in meal-dependent activation of brown adipose tissue [BAT (Habek et al. 2020)]. Interestingly, a recent study provided evidence that activation of BAT by uroguanylin applied intranasally is heavily influenced by age, gender, and phase of estrous cycle. Specifically, BAT activation was increased in older mice, reduced in female mice, and absent during estrus (Aleksandra Dugandžić, Zagreb). Therefore, metabolic studies, specifically those focusing on the regulation of BAT activity, should pay special attention to these parameters.

NO has long been known to cause cGMP-dependent vasodilation and cardiac relaxation. Impaired NO responsiveness in heart failure has been attributed to inactivation of NO by reactive oxygen species (Pacher et al. 2007). In contrast, HNO, which also is supposed to induce cGMP-dependent vaso- and cardiac relaxation, does not react with reactive oxygen species. Recently, the effects of DEA-NO and the nitroxyl donor Angeli’s salt were assessed in Langendorff-perfused hearts of rats with type 2 diabetes induced by high-fat diet and low-dose streptozotocin (Velagic et al. 2022). Whereas inotropic, lusitropic, and coronary vasodilator responses to DEA-NO were found to be impaired in type 2 diabetic hearts, responses to Angeli’s salt were preserved or even enhanced (Rebecca Ritchie, Melbourne). These results emphasize the potential of HNO donors in the treatment of acute ischemic attacks or heart failure.

Previous immunohistochemical analysis of murine heart revealed pericytes (PDGF receptor ꞵ-positive) and smooth muscle cells as NO-GC-expressing cardiac cells whereas neither endothelial cells nor fibroblasts stained positive for NO-GC (Friebe et al. 2018). One talk at the cGMP conference reported on NO-GC distribution in hearts of mice with cardiac hypertrophy induced by angiotensin II (Ang II) challenge (Lennart Kreutz, Würzburg). Here, NO-GC occurred in the developed fibrotic lesions as did the PDGF receptor ꞵ detected with an antibody or with a PDGF receptor ꞵ-td-Tomato-based lineage tracing Cre line. In addition, NO-GC colocalized with THBS4, a marker for activated fibroblasts, within the fibrotic lesions. The PDGF receptor ꞵ has been reported to occur in pericytes, smooth muscle cells, and some fibroblast subtypes. With additional td-Tomato-lineage tracing Cre lines specific for smooth muscle and for pericytes, respectively, both cell types were found not to migrate into fibrotic areas; hence, it can be concluded that expression of NO-GC is induced in activated PDGF receptor ꞵ-positive fibroblasts occurring in fibrotic regions during development of cardiac hypertrophy. Furthermore, analysis of cardiac function of WT and PDRF receptor ꞵ-specific NO-GC knockouts after Ang II treatment by pressure volume loops revealed lower cardiac output in the knockouts despite a similar degree of fibrosis as in wild type.

Another recent study analyzed the role of cGKI in cardiac myofibroblasts, the activated form of fibroblasts formed after Ang II challenge (Melanie Cruz Santos, Tübingen). The role of cGKI during Ang II-induced cardiac remodeling was addressed by a conditional tamoxifen-induced cGKI KO in activated cardiac fibroblasts (myofibroblasts) by means of Cre under control of the periostin promotor (mfKO). As periostin is known to be expressed upon phenotype change of cardiac fibroblasts to myofibroblasts during remodeling induced by Ang II treatment, cGKI was specifically deleted in myofibroblasts. Whereas cardiac hypertrophy did not differ between control and mfKO mice after prolonged Ang II treatment, the mfKO clearly showed an increased fibrosis and their myofibroblasts exhibited higher proliferation rate ex vivo. In accordance with an altered remodeling, mfKOs exhibited worsening of ejection fraction and fractional shortening. Thus, cGKI in cardiac myofibroblasts apparently attenuates the fibrotic response paving the way for potential therapeutic interventions targeting the cGMP/cGKI pathway to reduce or possibly reverse cardiac fibrosis.

A research group analyzed cGMP in the vicinity of cardiac mitochondria; furthermore, apoptosis of primary adult cardiac myocytes in culture was analyzed. Using a novel FRET-based biosensor targeted to the outer mitochondrial membrane, CNP and ANP-induced cGMP signals were shown to occur locally around the mitochondria. Activation of GC-A and GC-B with ANP and CNP, respectively, reduced apoptosis and PARP cleavage accompanied by reductions of caspase 9 activation and cytochrome c release. Mechanistically, this is ascribed to phosphorylation and inhibition of the proapoptotic DRP1 (Gaia Calamera, Oslo). Thus, natriuretic peptide-induced cGMP in the vicinity of mitochondria emerges as an antiapoptotic signal in cardiac myocytes.

(g) Natriuretic peptides and receptors

Determination of natriuretic peptides in plasma is important in the diagnostics of heart failure (Horiuchi et al. 2022; Mohan et al. 2023). One talk reported that processing-independent immunoassays for quantification of proANP, proBNP, and proCNP in plasma have been recently developed (Jens P. Goetze, Copenhagen). These immunoassays allow for quantification of pro-forms independent from endoproteolysis and peptide modifications.

CNP fulfills multiple roles in the cardiovascular system, in endochondral bone development, and during embryonic development (Moyes and Hobbs 2019; Ichiki et al. 2022; Straczynska et al. 2022), and more and more previously unknown functions are being discovered. At the cGMP conference in Augsburg, recent findings about a functional role of CNP/GC-B signaling in the lung were presented (Swati Dabral, Würzburg). A new mouse line with knockdown of GC-B developed exacerbated pulmonary hypertension under chronic hypoxia. In line with this observation, CNP reduced the proliferation and migration of cultured human pericytes. These data suggest that CNP/GC-B/cGMP signaling in the lung has protective effects to prevent the excessive vascular contraction and remodeling during pulmonary hypertension. Another talk reported that CNP regulates metabolic homeostasis (Cristina Perez-Ternero, London). A novel transgenic strain with inducible, global deletion of CNP exhibited reduced body weight, higher body temperature, and greater energy expenditure. Interestingly, CNP seems to exert metabolic regulatory actions in adipocytes by inhibiting sympathetic thermogenic programming via the G-protein coupled natriuretic peptide receptor (NPR)-C, whereas it drives adipogenesis via stimulation of GC-B. Hence, CNP exerts a pivotal role as a metabolic switch to balance energy homeostasis (Perez-Ternero et al. 2022).

The activity of the natriuretic peptide receptors is regulated not only by natriuretic peptides, but also by phosphorylation at specific sites of GC-A and GC-B (Potter 2011a, b; Kuhn 2016). Mutant mice were engineered in which GC-A or GC-B cannot be inactivated by dephosphorylation (Egbert et al. 2022), and recent results of studies with these mice were presented (Lincoln Potter, Minneapolis). One study revealed that preventing GC-B dephosphorylation rescues reduced axial and appendicular skeleton growth in a mouse model of achondroplasia, suggesting that dephosphorylation of GC-B transduces signaling pathways in bone (Wagner et al. 2021a). Another study demonstrated that increased phosphorylation-dependent activity of GC-A decreases cardiac hypertrophy and improves systolic function in male mice (Wagner et al. 2022). These data and other recent work established dephosphorylation as a mechanism to inactivate GC-A and GC-B, thereby limiting the effects of natriuretic peptides.

(h) Technological advances and new applications

The role of NO-GC-derived cGMP in the regulation of myocardial functions is still not well elucidated. Myocardial expression of NO-GC is barely detectable by immunohistochemistry, and FRET-based detection methods have provided conflicting results, showing either very low (Goetz et al. 2014) or no increases in cGMP (Menges et al. 2019) after NO stimulation of isolated cardiomyocytes. However, recently, it was shown that the co-culture of cardiac myocytes with cardiac fibroblasts allowed strong cGMP transients in cardiac myocytes to be monitored (Menges et al. 2019). Incubation with gap junction inhibitors reduced the cGMP fluorescence indicating fibroblast-to-myocyte-directed cGMP shuttle. To prove this concept, novel data were presented using a Tcf21-CreERT2 mouse, which expressed the cGi-500 FRET sensor (Russwurm et al. 2007) in cardiac fibroblasts (Michael Russwurm, Bochum). Furthermore, mice that expressed the FRET sensor specifically in cardiomyocytes were also used. Positive FRET signals in Tcf21-derived fibroblasts indicated that NO in fact activated NO-GC in this cell type to produce cGMP. Gap junction-mediated transfer of cGMP into cardiac myocytes was shown using acute cardiac slices from the cardiac myocyte-specific FRET mouse line. Further experiments showed that PDE3 was responsible for regulating cGMP levels in cardiac myocytes suggesting crosstalk between cGMP and cAMP. In fact, NO-GC activation led to increased cAMP response (induced by forskolin or isoproterenol) leading to enhanced phospholamban phosphorylation. By this mechanism, NO-mediated regulation of β-adrenoreceptor-induced cardiac contractility through cGMP/cAMP crosstalk could be possible. In vivo proof is, however, necessary to lay the foundation for this concept.

Activation of cGMP synthesis and subsequent measurement of cGMP levels have been reasonable methods to explore cGMP signaling in many cells and organs. However, subcellular compartmentation of cGMP signaling remains difficult to address due to the scarcity of targetable GC activators or sensors. The development of SponGee (Sponge inhibiting cGMP signaling) is a novel approach to explore cGMP-mediated communication. SponGee is a genetically encoded cGMP scavenger that interferes with cGMP signaling and subsequent pathways in individual cells (Ros et al. 2019). SponGee is a chimera based on fragments of cGK1α and cGK1β of the bovine cGMP-dependent protein kinase with medium affinity for cGMP (dissociation constant approx. 1 µM). Using the concomitant expression of a FRET sensor, SponGee was shown to prevent pharmacologically induced cGMP elevations, thus blocking downstream effects. High concentrations of cAMP did not influence the scavenging action of SponGee, underlining its specificity. Subcellular targeting of SponGee was provided using targeting sequences that restricted its expression to lipid rafts or to the plasma membrane outside lipid rafts (Lyn-SponGee: N-terminal fusion of palmitoylation-myristoylation targeting peptides from Lyn Kinase and SonGee-Kras: C-terminal fusion of a CaaX-polylysine motif derived from K-Ras, respectively). As proof of concept, using these constructs, the subcellular requirement of cGMP for the pathfinding of retinal ganglion cell growth cones was shown. Taken together, based on its subcellular resolution, SponGee is a novel tool to dig deeper into the compartmentalized world of cGMP signaling (Xavier Nicol, Paris).

## Outlook

The 10th anniversary meeting of the cGMP conference in 2022 was filled with novel insights not only into cGMP signaling but also in potential therapeutic approaches. After the introduction of a sGC stimulator for heart failure therapy following the tremendous success of a neprilysin inhibitor, the field seems currently expanding. There were also new data on novel compounds and treatment approaches of which some already started clinical testing. These preclinical and clinical data raise hope that in the future, cGMP research may lead to new therapies for diseases like neurodegeneration or nephropathies. Therefore, the whole community is looking forward to the next cGMP conference which will be held from June 28 to 30, 2024, in the hanseatic town of Lübeck in Germany.

## Data Availability

Review article/non-applicable.

## Abbreviations

ANP: Atrial natriuretic peptide
ARNI: Angiotensin receptor neprylisininhibitor
BNP: B-type natriuretic peptide
BA: Brown adipocytes
BAT: Brown adipose tissue
cAMP: 3′,5′-CAMP: cyclic adenosine monophosphate
cCMP: 3′,5′-CCMP: cyclic cytidine monophosphate
cGK: CGMP-dependent protein kinase, also called PKG
cGMP: 3′,5′-cGMP: Cyclic guanosine monophosphate
CNG: Cyclic nucleotide-gated ion channel
CNP: C-type natriuretic peptide
CNS: Central nervous system
CTEPH: Chronic thromboembolic hypertension
cUMP: 3′,5′-CUMP: cyclic uridine monophosphate
Cys: Cysteine
FRET: Fluorescence resonance energy transfer
GC: Guanylyl cyclase
GC-A: Guanylyl cyclase A
GC-B: Guanylyl cyclase B
GMP: Guanosine monophosphate
GTP: 5′-GTPG guanosine triphosphate
HF: Heart failure
HFpEF: Heart failure with preserved ejection fraction
HFrEF: Heart failure with reduced ejection fraction
KO: Knockout
MELAS: Myopathy, encephalopathy, lactic acidosis, stroke
NO: Nitric oxide
NO-GC: NO-sensitive guanylyl cyclase
NOS: Nitric oxide synthase
NP: Natriuretic peptide
PAH: Pulmonary arterial hypertension
PDE: Phosphodiesterase
PH: Pulmonary hypertension
tmGC: Transmembrane guanylyl cyclase, also called pGC
sGC: Synonym of NO-GC
SMC: Smooth muscle cell
SSc: Systemic sclerosis
WT: Wild type
